# A Dispersive Image Scanning Microscope for Multi-color High-resolution Imaging

**DOI:** 10.1021/acsphotonics.5c02836

**Published:** 2026-06-23

**Authors:** Lanna Bram, Ofir Tal-Friedman, Neta Fibeesh, Jasline Deek, Yohai Bar-Sinai, Eli Flaxer, Yael Roichman, Yuval Ebenstein, Jonathan Jeffet

**Affiliations:** † School of Physics and Astronomy, Faculty of Exact Sciences, 26745 Tel Aviv University, Tel Aviv 6997801, Israel; ‡ School of Neurobiology, Biochemistry and Biophysics, George S. Wise Faculty of Life Sciences, Tel Aviv University, Tel Aviv 6997801, Israel; § School of Chemistry, Faculty of Exact Sciences, Tel Aviv University, Tel Aviv 6997801, Israel; ⊥ Center for Physics and Chemistry of Living Systems, Tel Aviv University, Tel Aviv 6997801, Israel; # AFEKA—Tel-Aviv Academic College of Engineering, 69107 Tel-Aviv, Israel; ¶ Department of Biomedical Engineering, Tel Aviv University, Tel Aviv 6997801, Israel; △ Racah Institute of Physics, The Hebrew University of Jerusalem, Jerusalem 9190401, Israel; ▼ The Rachel and Selim Benin School of Computer Science and Engineering, The Hebrew University of Jerusalem, Jerusalem 9190401, Israel

**Keywords:** image scanning microscopy (ISM), confocal spinning disk (CSD), multi-color imaging, high-resolution imaging, fluorescence microscopy, spectral imaging

## Abstract

Fluorescence confocal microscopy is a fundamental and widely used tool for biological, medical, and chemical research. It enables color-coded visualization of sample components, enhancing our understanding of life’s building blocks and their interactions. The recent realization of image scanning microscopy (ISM) using confocal spinning disks (CSD) enhanced the spatial resolution of fluorescence microscopy to twice the diffraction limit with minimal sample perturbation and fast acquisition rates. However, capturing multicolor images using ISM is still time-consuming and introduces temporal artifacts as different colors are not acquired simultaneously. Here, we present a dispersive multicolor CSD-ISM system designed for concurrent enhanced-resolution and simultaneous multicolor acquisition. By integrating a custom linear Amici prism into the CSD-ISM optical detection path, we achieve multicolor, enhanced-resolution images at a fraction of the acquisition time and with a flexible color palette selection. A digital signal processor (DSP) is employed together with accompanying software as a cost-effective alternative to Field-Programmable Gate Arrays (FPGAs) used in previous studies. We provide an accompanying GPU-compatible, python-based image processing pipeline to decompose spectral signatures into multicolor channel-based images, while preserving the spatial resolution. Characterization using three color fluorescent beads demonstrated 1.74-fold resolution improvement and accurate color classification with a single simultaneous multicolor acquisition instead of the three acquisitions required in standard CSD-ISM. Application to neuron cells expressing a Parkinson’s disease-associated mutation, showcased improved resolution and contrast of four distinctly labeled cellular components. This multicolor CSD-ISM system provides a valuable tool for biological imaging, enabling the simultaneous acquisition of enhanced-resolution spatial information and multicolor data.

## Graphical abstract

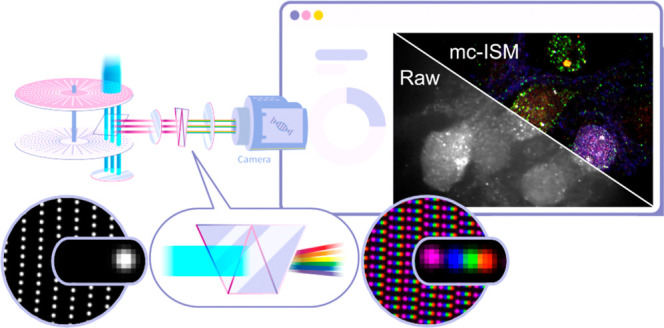

## Introduction

Science relies on fluorescence microscopy to visualize life at the smallest dimensions, enabling detailed visualization of cellular structures and dynamics. However, resolving intricate biological processes, especially within cells, necessitates observing the dynamic interactions between numerous molecules, often much smaller than the resolution limit (∼250 nm) of conventional optical microscopy. A common approach to enhance resolution is structured illumination microscopy (SIM),^1,2^ in which the sample is illuminated with a patterned excitation field at different positions and orientations while images are recorded with a conventional wide-field system. Thus, the illuminating patterns are used to extract additional spatial information and therefore increase the resolution up to 2-fold. Closely related to SIM is image scanning microscopy (ISM), originally conceived by Sheppard^3^ and later demonstrated experimentally by Müller and Enderlein.^4^ In ISM, a focused laser beam scans the sample, akin to confocal microscopy^5,6^ but instead of a single-point detector, a sensitive multipixel photodiode array or camera captures the emitted fluorescence, with its pixels functioning as an array of nearly zero-sized pinholes. This allows each pixel to simultaneously act as both a confocal pinhole and a detector, collecting the spatial distribution of emitted light around each illuminated point. The key innovation in ISM lies in the “pixel reassignment” process,^3^ where detected light is reassigned to its correct spatial position, effectively narrowing the point spread function (PSF) and improving resolution. This reassignment process with sufficient data sampling allows reconstructing a super-resolved image, achieving a 2-fold improvement in lateral resolution compared to conventional, diffraction limited, confocal microscopy.

However, the standard scanning confocal ISM is notably slow. To address this Schulz et al.^7^ implemented ISM in combination with confocal spinning disk (CSD),^5,6^ increasing image acquisition speed by nearly 100 times. By replacing the single pinhole with two high-speed rotating disks featuring hundreds of pinholes and matching microlenses, CSD generates numerous simultaneous excitation volumes to scan the sample rapidly. Integrating over the entire disk rotation generates homogeneous illumination, with high contrast and Z-sectioning capabilities. In CSD-ISM, the integration over the continuous disk rotation is replaced by synchronized stroboscopic excitation at predefined angles to generate a precise patterned illumination. To reconstruct high-resolution images, the reassignment algorithm first localizes the excitation spots in each frame using reference data from a homogeneously fluorescent sample. To correctly integrate all the frames into a high-resolution image, the algorithm then reassigns the information from each pixel halfway back toward the center of the corresponding excitation focus, thereby correcting the inherent optical offset. Computationally, this is efficiently implemented by registering raw data into a refined image grid with twice the pixel density in each dimension. The accumulation of these reassigned signals effectively shrinks the system’s PSF, doubling the lateral resolution.^7,8^ Consequently, the technique has become widely adopted in medical and biological research with commercial implementations of the technique available. Microscope modules such as SoRa (CSU–W1 SoRa Confocal Scanner Unit, Yokagawa)^8–11^ and IXplore SpinSR (IXplore IX83 SpinSR, Olympus)^12–14^ offer integral doubling of lateral resolution as their standard confocal module. Consecutively, methods such as iSIM^15^ and CSD-OPR^16^ were introduced, where hardware-based all-optical photon reassignment allows bypassing the computational demand, achieving up to 100 fps with analog in-acquisition processing.

While high resolution is crucial, in fluorescence microscopy color information is equally important, providing essential contrast between different entities within the sample. Conventionally, multicolor imaging is established by sequential acquisition with different filters. In CSD-ISM, this leads to a significant extension of acquisition time, as hundreds of frames are needed in each channel to reconstruct the high-resolution image. Furthermore, this results in spatiotemporal chromatic artifacts, as different colors are recorded at different times.^17^ Alternatively, split-view setups^18^ allow for registering several colors simultaneously, albeit at the cost of a reduced field of view size and the requirement for cross-channel registration. More recently, spectral imaging was introduced,^19–24^ enabling the recording of color information unrestricted by discrete color channels. Spectral information is recorded by dispersing the detected light onto a different camera or a part of the camera. This, however, restricts throughput due to the large spectral dispersion induced, smearing each PSF over a large pixel area, resulting in a relatively small effective FOV^25,26^ (for further examples see Table S1 in^27^). Recently, compact PSF engineering for spectral determination^27–33^ has emerged. Methods such as continuously controlled spectral resolution (CoCoS) allow continuous tuning of the spectral dispersion for optimized performance.^27^ These methods distribute color information locally, registering simultaneously all color information with higher throughputs and fluorophore densities.^27–30^ Implementing multiplexed multicolor acquisition with multifocal ISM has been recently pursued by multifocal scanning microscopy (MSM), demonstrating simultaneous dual-color super-resolved acquisition using a stationary interleaved dual-color multifocal excitation. Instead of moving the excitation pattern through the sample as in CSD-ISM, in MSM the stage is used to scan through the sample while the excitation remains stationary.^28^ However, this method is limited both in color-multiplexing capability, requiring to finely align the different diffraction pattern lattices for each color, and in achievable imaging speed, limited by the stage motion and accuracy.

Here, we combine CSD-ISM with compact multicolor registration (Figure 1). CSD-ISM, capturing a sparse array of confocal spots in each frame (Figure 1b, left panel), offers a unique advantage for integrating multicolor registration. By using a prism placed in the detection path (see Figure 1a) to disperse the spectral information contained in these spots, we encode all colors in a single CSD-ISM acquisition, better exploiting the full FOV of the camera (Figure 1b, right panel). This novel approach provides a powerful tool for high-resolution, multicolor imaging, enabling the simultaneous capture of spatial and color information by using a standard CSD-ISM microscope with minimal modifications.

**Figure 1:**
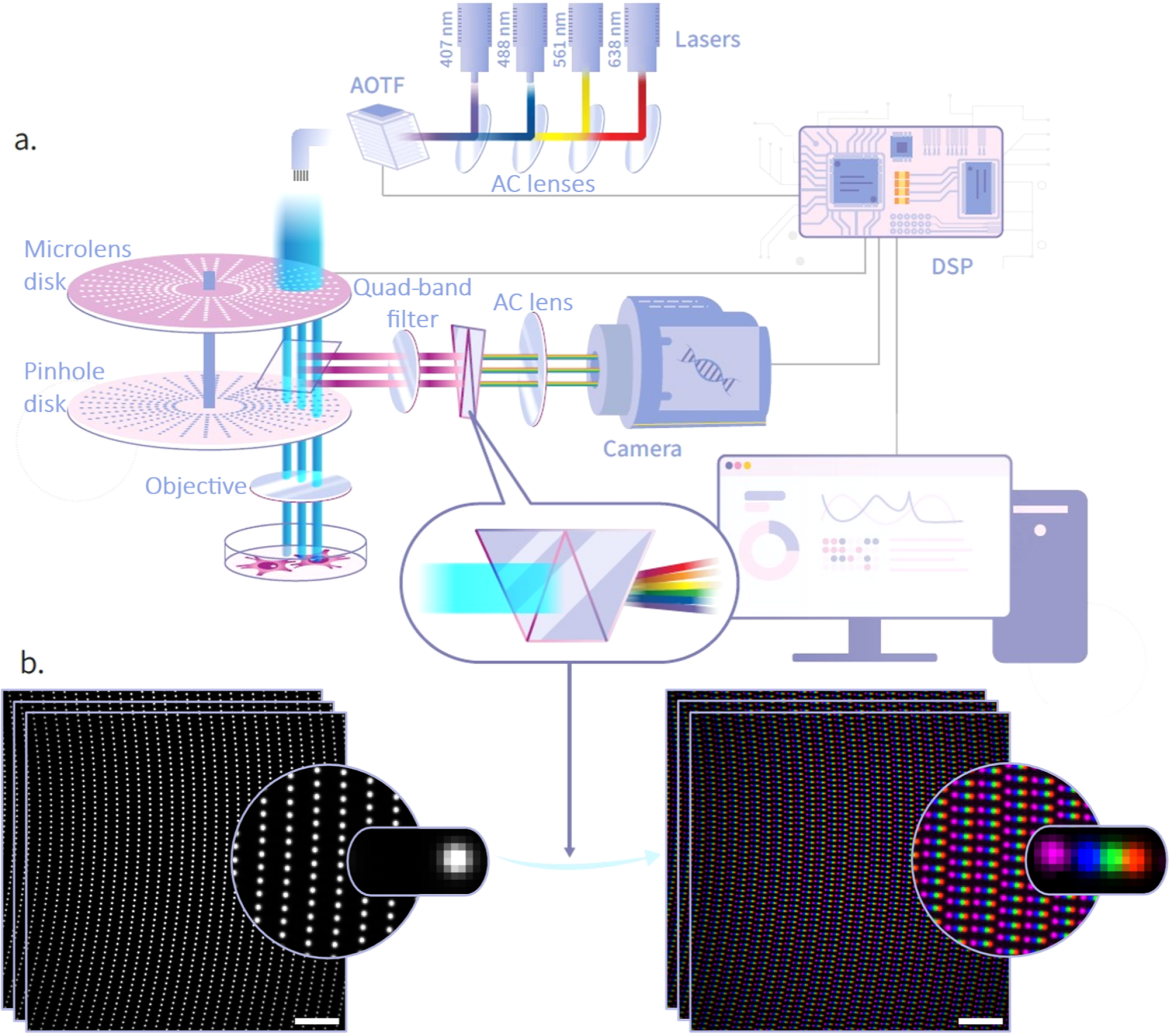
Schematic representation of the multicolor confocal spinning disk image scanning microscopy (CSD-ISM) system. (a) Optical setup. Multiple laser lines are combined and controlled by an Acousto-Optic Tunable Filter (AOTF) to selectively excite fluorescence. The excitation light is directed through a spinning disk system comprising microlens and pinhole disks. The emitted fluorescence signal is collected through a polychroic mirror and a notch emission filter and passed through a custom Amici prism, which disperses the fluorescence emission into its spectral components and onto the camera. For the stroboscopic illumination required for ISM implementation, a digital signal processor (DSP) reads the disk’s spinning frequency and sends pulsed signals to the AOTF and camera, synchronizing the laser pulses and camera exposures to the spinning disk frequency. (b) Example imaging output from the system. Image acquisition consists of several frames, each capturing a scanned sample image at a specific rotation angle of the disk. Summing all frames reconstructs the full image. The left panel shows raw images scanned without a prism, showcasing the structured illumination pattern induced by the disks. The right panel shows the result of dispersing colors using the custom amici prism. Each color is translated along the horizontal axis, capturing its spectral information. For illustration purposes, a false-colored, overlaid, multisample, and multiacquisition (Alexa 405 in purple, Alexa 488 in blue, Alexa 568 in green and Alexa 647 in red) image was acquired using the custom prism (actual multifluorophore samples would have been recorded in a single grayscale manner). Scale bars are 10 μm.

## Principles

### Optical Setup

The optical setup was designed and constructed by combining dispersive-color registration and ISM with a standard commercial CSD (Figure 1a, for further details see “Materials and Methods”). To record high-resolution images, the stroboscopic illumination required for implementing ISM was controlled using a custom designed digital signal processor (DSP),^34,35^ synchronizing the spinning disks’ rotation frequency with the camera exposure and laser illumination. The acquisition protocol was designed following the protocol outlined by Qin et al.,^36^ offering a cost-effective solution compared to the previous FPGA implementation (more than 70× price reduction^34^). A custom Amici prism was introduced at the output of the commercial CSD to register color simultaneously. Amici prisms, also known as direct vision prisms, disperse colors without deviating the optical axis,^27,33^ allowing to conveniently place the prism in the emission path without requiring any additional optics. Unlike standard prisms which disperse shorter wavelengths more strongly, we specially designed the prism to linearly disperse the visible spectrum (Section S1 and Figure S1) in the vicinity of the CSD spots. The linear dispersion, which was validated experimentally using homogeneous fluorescent samples (Section S2 and Figure S2), offered better exploitation of the area between illumination spots essential for maximizing color content. The integration of the prism results in the spectral dispersion of the fluorescence emitted from the sample, allowing us to encode each color into a unique spatial intensity distribution, which we termed as the “spectral PSF”. Therefore, each confocal spot reports both on the fluorescence intensity and the underlying fluorescent markers composition in its diffraction-limited volume (Figure 2a).

**Figure 2:**
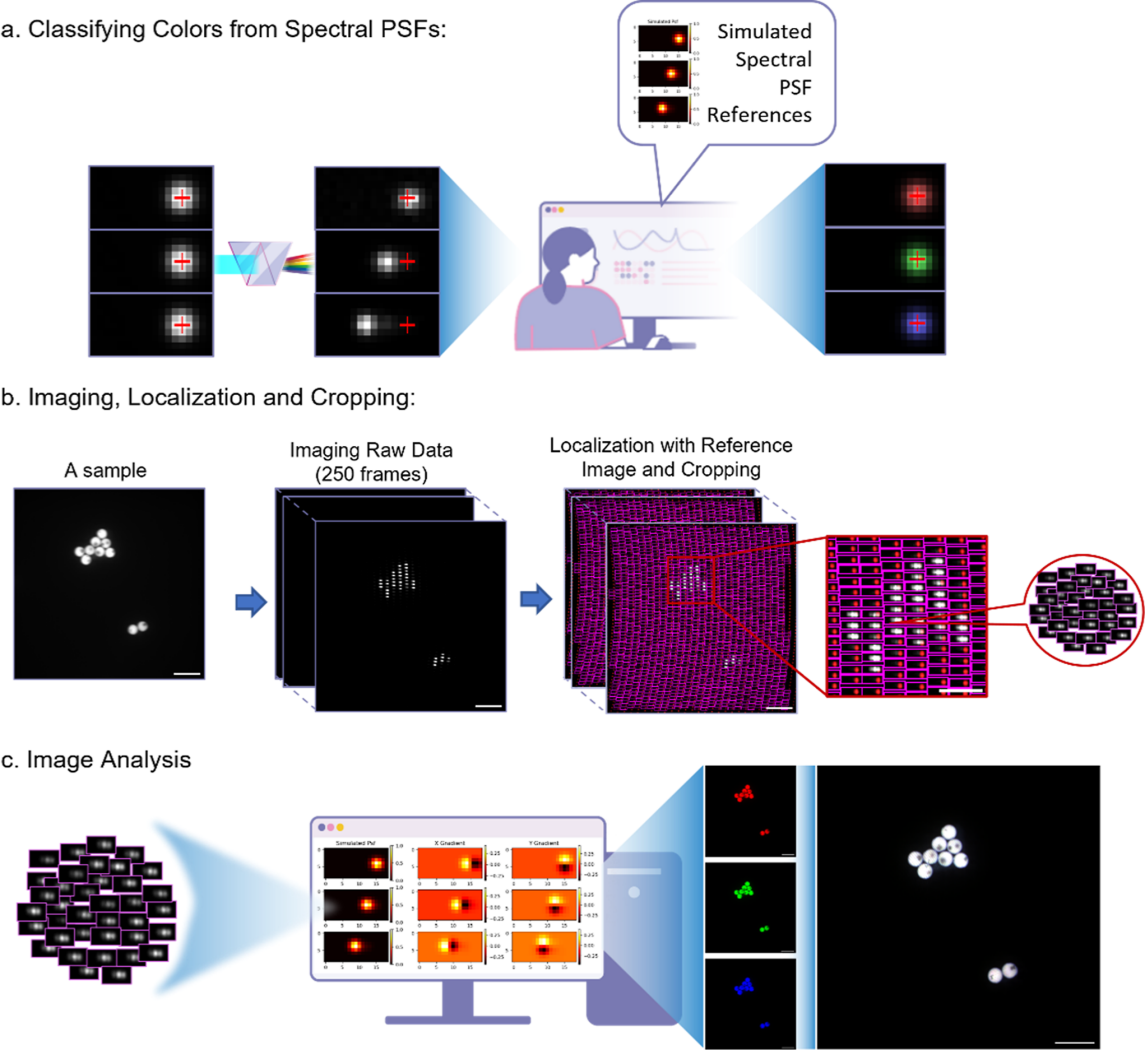
Workflow of CSD-ISM image processing, localization, and analysis. (a) Schematic illustration of color classification. Left: three simulated examples of confocal volumes containing three distinct colors without dispersion. Red crosses correspond with the confocal illumination reference localization. Center: a prism displaces each color with a distinct distance compared to the illumination reference, providing a unique spectral signature. Right: using linear regression with simulated spectral PSFs, the underlying color composition in each confocal volume is resolved and assigned to the nondispersed location. (b) Imaging, localization, and cropping process. Left: Experimental image of three-colored 4 μm fluorescent beads without dispersion. Center: A stack of 250 spectrally dispersed frames captures the full information content of the sample. Right: A reference stack of the illumination profile (in red) is used to crop spectrally dispersed ROIs around each illumination volume across all frames. (c) Image analysis and spectral decomposition. Left: The spectrally dispersed crops are decomposed into individual color channels using linear regression based on the simulated spectral PSFs and their gradients, and are spatially reassigned with the ISM algorithm. Right: The final output is a high-resolution multicolor nondispersed color image. Scale bars are 10 μm.

### Image Analysis

To accurately link fluorescence wavelengths to color readouts, establishing a conversion function between fluorescent markers and their spectral PSFs is essential. This can be achieved through experimental imaging of homogeneous single-marker samples to register each of their spectral responses or, more efficiently, via spectral PSF simulations.^33,34^ To create a conversion function which can translate between spectral PSF and its underlying fluorescent markers composition, we created a calibration curve using multiple homogeneously distributed single-marker fluorescent samples with emission peaks across the visible spectrum (Figure 1b as an example. For details, see Methods and Figure S2). For each sample, the spectral PSF’s center of mass was fitted with its corresponding emission spectrum’s center of mass after accounting for the spectral response of the camera and filters (Figure S2a). This allowed us to establish a calibration model between spectral wavelength and pixel displacement due to dispersion, enabling the accurate simulation of any fluorescent spectral PSF according to its theoretical emission spectrum (see Figures S2–S4 and Section S3). The resulting simulations not only validated the experimental data but also extended to unmeasured fluorophores, enhancing the versatility of the imaging system and serving as the basis for our downstream linear regression color classification model. Thus, this simple calibration, requiring no more than four samples across the spectrum, enabled us to extend our color palette and resolve fluorophores without the need for experimental registration of their spectral response.

After establishing the spectral response of our optical system, to capture multicolor high-resolution images, the frame-stacks of spectrally encoded confocal spots need to be deciphered both spatially, using the ISM reassignment algorithm, and spectrally, converting the registered spectral signals into nondispersed color-channel-assigned signal.

The ISM reassignment requires precise knowledge of the confocal spots’ illumination pattern with subpixel resolution at each frame.^3,36^ To register their position, we used a reference sample of dense homogeneously distributed fluorescent far-red dye (Alexa Fluor 647—AF647), localizing and registering all illumination spots’ subpixel positions in each frame (Figure 2a,b) using our open-access GPU-enabled python image analysis pipeline: Spectral Extraction and Classification for ISM Analysis using Least Squares (SPECIALS—https://github.com/ebensteinLab/SPECIALS). With a single calibration acquisition, we could easily implement the ISM algorithm on all samples acquired that day (see Methods).

The color-from-spectral reassignment was carried out using the same reference. Essentially, each illumination confocal spot can excite a linear combination of fluorophores found within its confocal volume. Therefore, we used linear regression to estimate the linear combination of spectral PSFs imaged around each registered location in the reference. In practice, we crop a rectangular region of interest (ROI) from the sample’s data around each of the registered illumination localizations (Figure 2b). For each ROI we estimate its underlying fluorophore composition by comparing it to a linear combination of simulated spectral PSFs according to the dyes used to label the sample. Finally, we assign the extracted linear coefficients to a multichannel nondispersed PSF, creating a multichannel color representation of the ROI positioned at the reassigned spatial location according to the ISM algorithm (Figure 2a,c).

One key principle underlying the resolution enhancement of ISM is the parallax effect when imaging the same sample from different angles. Small paraxial spatial shifts induced by exciting and imaging the sample through a variety of microlens positions and viewing angles (see Section S3, Figure S3), allow with the correct signal reassignment to double the resolution. To approximate the parallax-induced spatial shifts during our color assignment, we extracted the first term coefficients of the two-dimensional Taylor expansion by adding the spectral PSFs’ gradients to the linear regression model (Figure 2c center, detailed explanation in Section S3). This allowed us to maintain the spatial resolution during the color reassignment with only a slight deterioration (Figures 2c, S5 and Section S3). A beneficial feature of the linear regression analysis used for color assignment is that it is calculated by a closed formula without the need for optimization of the coefficients. This allows calculating the color compositions of millions of cropped ROIs in a fraction of a second on the CPU, keeping the computation time on par with the imaging acquisition time.

After each ROI was reassigned both spatially and spectrally, the individual ROIs are summed and reconstructed to create a multichannel composite image of the sample (Figure 2c, right panel). Finally, for resolution enhancement, ISM reconstruction and further deconvolution were applied. A flowchart scheme of the full image analysis pipeline is presented in Figure S6.

## Results

### Multicolor CSD-ISM Characterization and Validation

To benchmark the color and spatial resolution of our multicolor CSD-ISM system, we imaged a mixture of three samples of single-colored, subdiffraction-limit, fluorescent beads (170 nm diameter, emission maxima at 515 nm, 560 and 660 nm for the blue, green, and red beads respectively) (see “Methods” section for details). The results presented in Figure 3 show good assignment of colors, and a clear improvement in the resolution of the ISM and the Richardson-Lucy (RL) deconvolved^37,38^ ISM images (Figure 3a–c) (see “Methods” for a description of the deconvolution process). To quantify the improvement in resolution, we measured the full width at half-maximum (fwhm) of the red beads. These beads, unlike the other colors, presented almost no apparent dispersion in our system (see Section S3, Figure S4), thus allowing a direct comparison between images pre- and post-color and ISM reassignment. Our calculation (Section S4, Figure S7) shows a 1.74 ± 0.24 -fold resolution enhancement of the deconvolved color ISM image compared to the sum of raw spectral images. As shown in Figure 3c, resolution enhancement is visually apparent in all channels, albeit an evident slight resolution deterioration due to the Taylor approximation used in the color assignment. Trying to better account for larger spatial parallax shifts by introducing the next order in the Taylor approximation (second derivatives) to the linear least-squares regression did not improve the results (see Section S3, Figure S5). Nonetheless, the ISM reconstruction and deconvolution significantly improved spatial resolution, overcoming this initial loss. Consequently, a single acquisition of 500 frames provided a high-resolution three-color image, eliminating the need to make multiple multiframe acquisitions for each color separately as in standard CSD-ISM.

**Figure 3:**
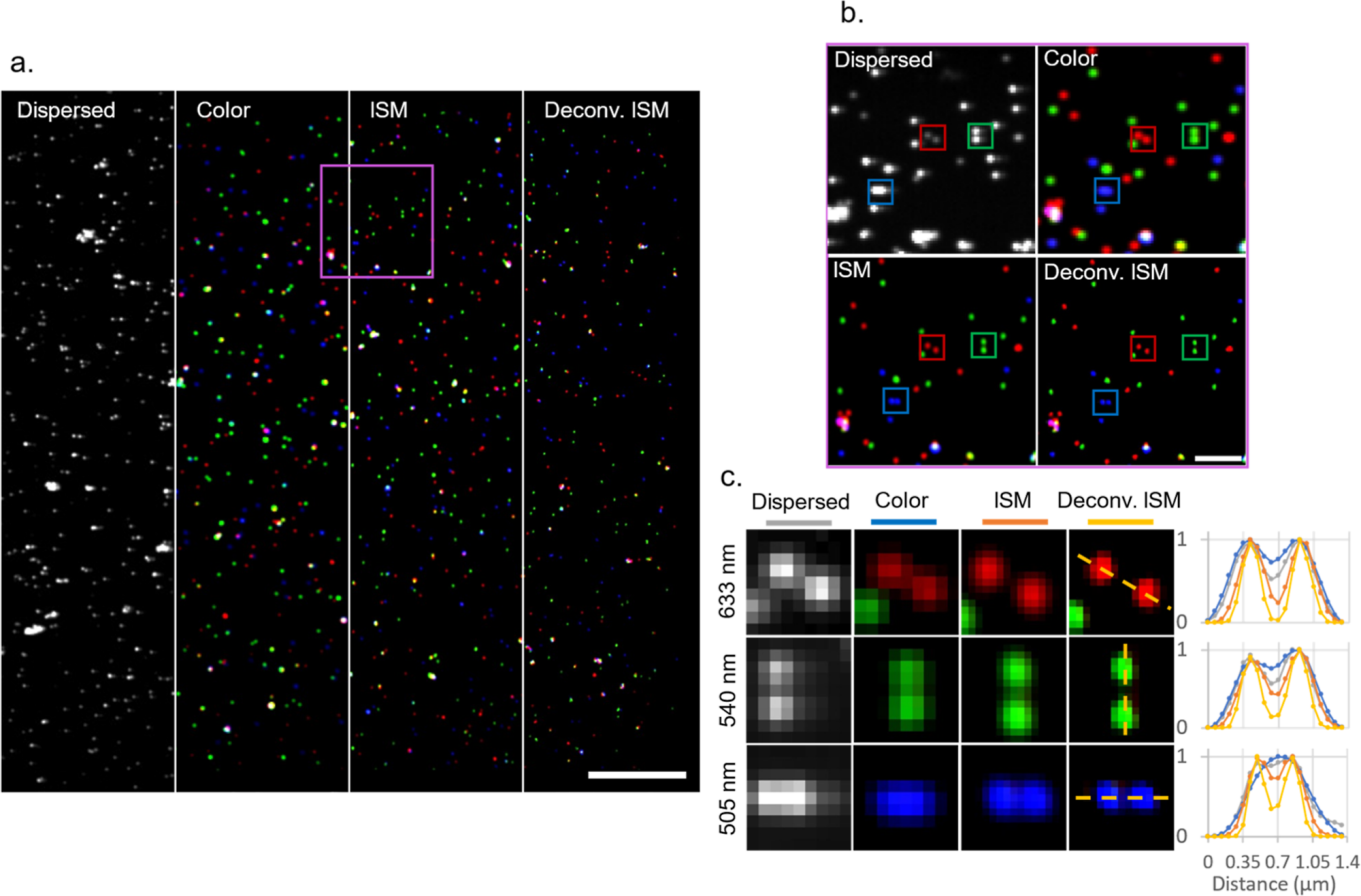
Multicolor ISM validation with fluorescent beads. (a) Widefield image of 170 nm diameter fluorescent beads with emission maxima at 515 nm (blue colored), 560 nm (green colored) and 660 nm (red colored) at different stages of processing. The different sections of the image showing: an average of 500 spectrally dispersed frames (dispersed), color-assigned no-dispersion image (color), color ISM image (ISM), and the final deconvolved ISM image after applying RL-deconvolution (Deconv. ISM). Scale bar: 10 μm. (b) Magnified view of the magenta rectangle highlighted in panel (a), demonstrating the resolution enhancement. Rectangles mark zoomed-in regions shown in panel c. Scale bar: 2 μm. (c) Representative examples of pairs of beads of different colors at each processing stage. At the right side, normalized intensity profiles of the bead pairs along the dotted yellow lines.

### Biological Samples

To showcase our method in a biologically relevant scenario, we imaged neuron cells expressing a mutant a-synuclein (α-Syn) associated with familial Parkinson’s disease.^39^ Neurons were transduced with an adeno-associated virus consisting of the mutation and a green fluorescent protein (GFP) label to investigate specific downstream consequences of pathological α-Syn on proteins of interest. Such proteins include the RNA binding protein G3BP and the synaptic protein synaptotagmin-1. G3BP marks stress granules which can range in sizes from 100 nm to several micrometers^40^ and synaptotagmin-1 is a key component of synaptic vesicles which can be as small as 40 nm.^41^ Improved visualization and deconvolution of these structures can facilitate enhanced understanding of the localization, interaction and aggregation of these proteins. Apart from the endogenous GFP, immunofluorescence was used to fluorescently label the proteins G3BP and synaptotagmin-1 with CF568 and CF647 secondary antibodies, respectively. DAPI was used to stain DNA in the nuclei. Thus, Figure 4 presents a simultaneous four-label imaging with a single acquisition of 250 frames. This demonstration showcases the four-color assignment and spatial resolution improvement, revealing finer cellular structures (panels b,c). Nonetheless, some image artifacts are visible in the reconstructed images, caused by inherent optical misalignment in our system (see Section S5 and Image S9).

**Figure 4:**
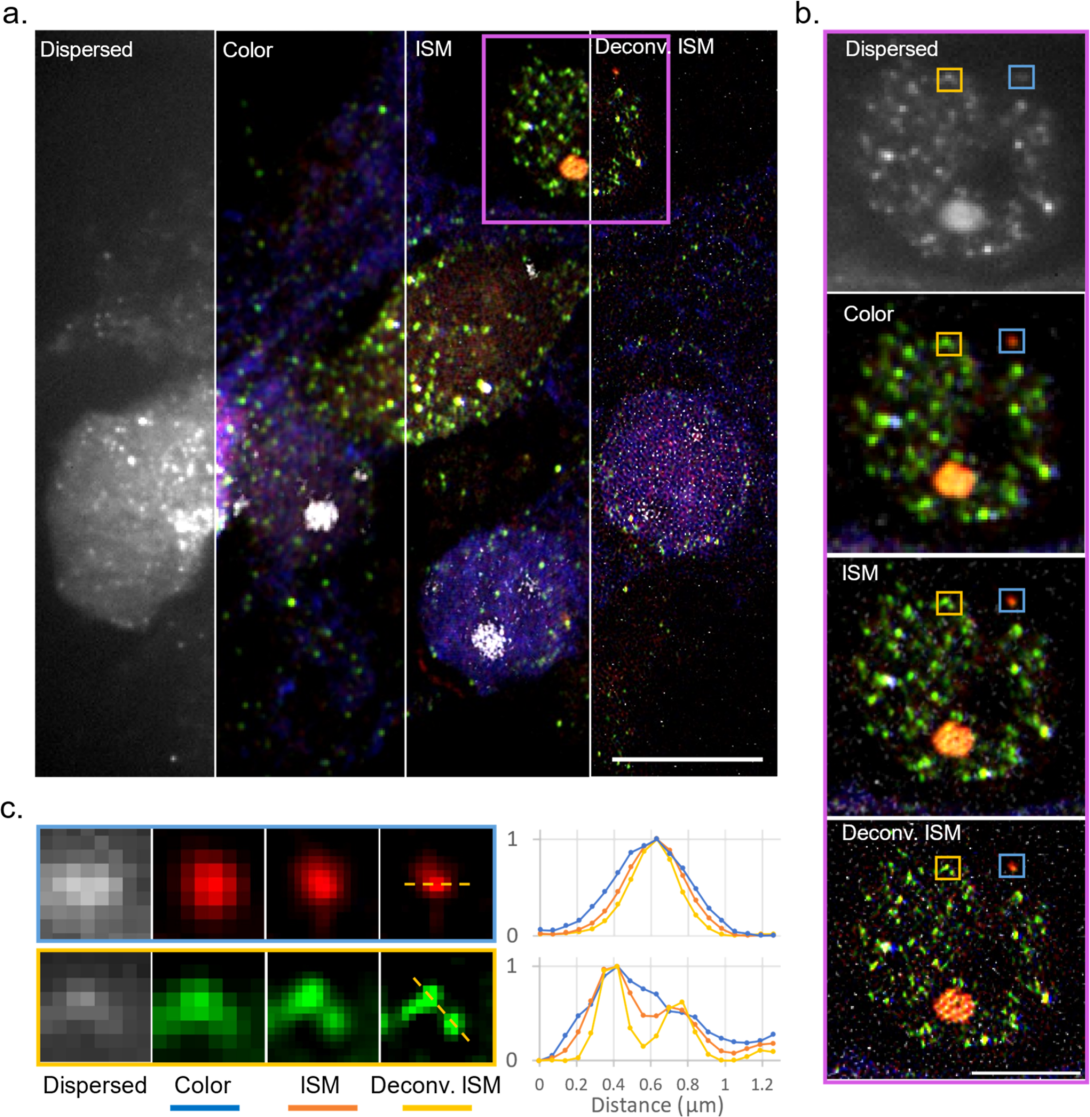
Multicolor ISM imaging of biological cells. (a) Widefield images of biological cells at different processing stages: sum of 250 spectrally dispersed images (dispersed), sum of color assigned image without ISM reassignment (color), color assigned with ISM pixel reassignment (ISM), and RL-deconvolved spectral ISM image (Deconv. ISM). Color code: white—DAPI stained DNA in the nuclei; blue—endogenous expression of eGFP by adeno-associated virus; green—CF568 labeled G3BP RNA binding protein; red—CF647 labeled synaptotagmin-1 synaptic protein. Scale bar, 10 μm. (b) Zoomed-in view of selected cellular region, as indicated in (a). Scale bar, 5 μm. (c) Zoomed-in views of selected regions in (b) in the red (top row) and green (bottom row) channels, with corresponding normalized intensity profiles along the dotted yellow lines.

## Conclusions and Discussion

In this study, we have successfully developed and validated a novel multicolor CSD-ISM system that combines high-resolution imaging with multicolor acquisition, enabling the simultaneous recording of spatial and color information in biological samples. By integrating a custom-designed linear Amici prism into a CSD-ISM setup, we achieved efficient multicolor encoding and improved resolution, as demonstrated through imaging of fluorescent beads and neuron cells.

To convert the acquired spectral information into standard color-channel readout, we have established a comprehensive pipeline (see flowchart in figure S6) coded in python and openly available via github (https://github.com/ebensteinLab/SPECIALS). The experimental protocol requires two calibration steps: first, a one-time experimental registration and calibration of the system’s spectral response, allowing us to computationally extend the spectral readout to any fluorescent marker with known emission spectrum; and second, an experimental illumination pattern registration, recorded once per day, to accurately calibrate the ISM algorithm. With these two calibrations, the full experimental spectral data stack (acquired in one-go) is segmented into ROIs, which subsequently undergo spectral-to-color reconstruction using a fast linear least-squares regression. The reconstructed color-channel ROIs are then summed using the ISM pixel-reassignment algorithm creating a high-resolution color-channel image.

Crucially, the custom-designed prism makes the system adaptable to users’ choice of fluorescent markers and filters. The prism linearly disperses colors within the visible spectrum, encompassing the emission spectra of most available fluorophores and fluorescent proteins. Consequently, a one-time spectral calibration using four homogeneously distributed samples is sufficient to fully characterize the system’s spectral response. This step allows users to computationally simulate the spectral PSF of any fluorophore accounting for the system’s filters spectra, rendering the setup highly modular without requiring physical reconfiguration. To further enhance this modularity, future iterations could integrate CoCoS microscopy,^27^ allowing users to dynamically tune the spectral dispersion and optimize performance and spectral separation between markers. This will be useful where optimized spectral resolution is required to distinguish between specific markers of choice.

Our system characterization using multicolored 4 μm fluorescent beads confirmed the accurate color representation and validated the image reconstruction process. Furthermore, imaging of 170 nm (subdiffraction-limit) fluorescent beads demonstrated a 1.74-fold resolution improvement from the spectral image to the deconvolved multicolor ISM image, highlighting the effectiveness of our approach in enhancing spatial resolution. Our method offers a boost to the image acquisition speed since it enables simultaneous imaging of all channels, rather than capturing individual color-channels sequentially. However, since our linear regression model is limited in registering large parallax-induced lateral translations, we observed a slight decrease in lateral resolution. As discussed in Section S3, further improvements to the resolution could be introduced by improving the regression analysis, thus accounting for larger parallax translations without interfering with color assignment. Nonetheless, our approach offers a good balance between performance and resolution, allowing us to analyze millions of spectral ROIs in a fraction of a second on the CPU with only a slight deterioration of resolution (Figure S8). Maintaining a fast calculation is advantageous as it allows for a future implementation as an on-the-fly process, where the color decomposition is calculated alongside with the spectral image acquisition.

Another limitation of our current process is the requirement for nonoverlapping spectral PSFs for accurate color decomposition; samples must be prepared with fluorophores who’s spectral PSFs are separated by at least the fwhm of the PSFs, to ensure correct color differentiation while accounting for the parallax shifts. Although this is a standard requirement in fluorescence microscopy to avoid bleed-through between channels, we have strong evidence^32,33^ that future works using machine learning could exploit subtler nuances of the spectral PSF, thus overcoming this limitation.

The application of multicolor CSD-ISM to imaging neuronal cells from a Parkinson’s disease study showcased the system’s ability to resolve finer cellular structures and enhance image contrast. However, some artifacts, commonly reported in CSD-ISM imaging, were observed in our images, likely due to low SNR and optical misalignment in our CSD system. Previous studies by the Enderlein group have characterized such artifacts both theoretically and experimentally.^36,42^ These artifacts have been attributed to under sampling of scan positions, which can occur due to a limited number of frames, low signal-to-noise ratio (SNR) in either the reference or spectral image, optical misalignment or inaccurate up sampling factor in ISM reconstruction. By comparing the results of acquisitions with a larger number of frames (1000 instead of 250) and increased up-sampling ISM reconstruction factors (up to 16), we were able to rule out these potential sources of artifacts. However, due to optical misalignments in our CSD, our baseline images showed nondiffraction limited performance, with PSF’s fwhm showing ∼1.9 larger spread than expected even without the prism installed (see Section S6). Moreover, due to excitation laser misalignment, resulting in low excitation power at the sample and therefore low SNR, it was not possible to eliminate these artifacts completely (see Figure S9). Moreover, we observed that these artifacts already appeared in the color decomposition images, prior to ISM reconstruction, which points toward an uneven sampling of the sample space by our excitation spots profile. We therefore conclude that these artifacts could be addressed by optimizing hardware alignment.

Moreover, future implementation of advanced image reconstruction algorithms can computationally mitigate these misalignments and therefor reduce artifacts. For example, Adaptive Pixel Reassignment (APR)^43^ inherently corrects for local optical aberrations by dynamically estimating spatial shifts directly from raw data via cross-correlation (in contrast to the rigid half-distance pixel reassignment approach used here). Alternatively, Autocorrelation Inversion^44^ provides a blind reconstruction method that bypasses the need for prior PSF knowledge, isolating high-frequency structural details directly from spatial autocorrelations. Implementing these algorithms would further enhance spatial resolution and system robustness, though at the potential cost of higher computational complexity and rendering times compared to our fast OLS approach.

To summarize, this integrated approach offers several advantages. First, it shortens and simplifies the process of acquiring multicolor images, eliminating the need for color alignment by computationally intensive postprocessing. Second, it leverages the inherent high-resolution capabilities of CSD-ISM, allowing for the visualization of fine details within biological samples. Finally, it is readily implementable in any lab equipped with a CSD system, making high-resolution multicolor imaging more accessible to a broader range of researchers. Overall, the multicolor CSD-ISM system presented here offers a powerful tool for multicolor, high-resolution imaging of biological samples. Future work will focus on optimizing the system for improved SNR, reducing artifacts, and expanding its capabilities for 3D imaging and dynamic live-cell applications.

## Materials and Methods

### Experimental Setup

Initially, we reconstructed the CSD-ISM system much like the protocol outlined by Qin et al.^36^ The essential components of our CSD-ISM include an Olympus IX71 Inverted Research Microscope implemented with a 100×, 1.40 NA objective lens (Olympus UPlanSApo 100×, Oil) that was controlled by a piezo (E–665 Piezo Amplifier/Servo Controller), Andor Precision Control Unit ER-PCUB-110, and Yokogawa Confocal Scanner Unit CSU-X1. As a light source, we used Andor Laser Combiner ALC-501A, with laser wavelength and power modulation via an AOTF that contained four lasers- 407 nm (Coherent CUBE), 488 nm (Sapphire Coherent Laser), 561 nm (Cobolt Jive) and 638 nm (Cobolt 06-MLD). Throughout all measurements, the lasers were operated with a laser power at the sample plane of 0.02 mW, 0.45 mW, 0.22 mW and 0.5 mW respectively, corresponding to 0.4 W/cm^2^, 9.2 W/cm^2^, 4.5 W/cm^2^ and 10.2 W/cm^2^ excitation power densities. A multiband filter (440/521/607/700 nm BrightLine quad-band bandpass filter) was used in the detection path of the CSD. For dispersion, a custom linear Amici prism (see details below) was used and integrated between the CSD and the camera. Final images were collected by an Andor iXon+ 897 EMCCD Camera. For hardware synchronization, we used a DSP-based controller integrated with a Windows-based control software (see details below).^34^

### DSP-based Controller

For hardware synchronization in our multicolor CSD-ISM system, we implemented a DSP. Based on the methodology in,^34^ the DSP was selected as a cost-effective and accessible alternative to FPGA solutions. The DSP synchronizes the CSD frequency with the laser pulse (managed by an AOTF) and the camera shutter. The DSP receives pulses from the CSU as a reference and then generates two digital waveforms: one for the camera shutter and the other for laser activation, with each rising edge of the input pulse initiating a new scan cycle. Furthermore, a Windows-based application was provided for simplified control of the system’s triggering hardware. This software manages eight digital parameters and four analog laser intensity values through a custom communication protocol.

The parameters are.

- TC_ON_: spinning cycle per frame
- TC_OFF_: spinning cycle for camera readout
- TL: laser pulse width (μs)
- TR: laser pulse rate (μs)
- TD: frame shift time (μs)
- TA: camera activation time (μs)
- LPN: laser pulses per SSC (single scan cycle)
- N: total Frames’ Number

The DSP calculates a dynamic delay time (TK) for each frame using the formula: TK = TA + K*TD, *K* is the frame index, ensuring accurate temporal coordination during image acquisition. Furthermore, it also monitors the rotating disk frequency, enforcing adherence to predefined parameter limits. An automated rate function measures the disk frequency, calculates TR and TD values, and automatically configures the hardware accordingly. To evaluate the effectiveness of CSD-ISM with this DSP, we benchmarked it with a sample of diffraction-limited fluorescent beads and confirmed a resolution enhancement of 2.11×, in accordance with Ref 36 (see Figure S10). For three-dimensional (3D) imaging, further settings need to be established, including the total number of layers for 3D imaging, specifying the interval between layers, and setting the initial position (Figure S11). The application and its communication library were developed using National Instrument LabWindows/CVI, ensuring compatibility with ANSI C and the Win32 API.

### Prism Design

To extend the multiplex capabilities of our system we designed a custom compound double Amici prism. Amici prisms retain the optical axis for a central wavelength while light of shorter or longer wavelengths is dispersed in opposite directions. We optimized our prism design using Zemax^45^ for distinguishing between five spectral channels by exploiting the available pixel area between adjacent CSD excitation spots (Figure S1). To efficiently encode all spectral channels, we designed the prism’s dispersion to have a linear wavelength dependence, ensuring homogeneous spectral resolution for the entire visible spectrum and therefore optimized multiplexing (additional information on the optimization and design is given (Section S1). Based on this optimization, a linear Amici prism was manufactured (Shanghai Optics) and integrated into our setup with a custom built, light-tight holder at the output of the CSU-X1.

### Sample Preparation

i Spectral calibration—Homogeneous emission across the field of view is essential for acquiring reference images for calibration at different wavelengths. We used a variety of dyes each dissolved in DMSO to 10 mM concentrations (Alexa Fluor (AF) dye 405-DBCO, AF488-DBCO, AF532-DBCO, AF546-DBCO, AF568-DBCO, AF647-DBCO, and Cy5.5-DBCO, Jena Bioscience, Jena, Germany), and diluted at 1:100 volume ratio with TE buffer (10 mM Tris and 1 mM EDTA (pH 8)). Eight μL of the resulting solution was placed between a prewashed microscope slide and a standard 22 × 22 mm^2^ coverslip in order to create a homogeneous dye solution film for each color.
ii Image analysis pipeline—we used a commercially available test slide containing 4 μm multicolored fluorescent beads, stained with blue (350/440 nm), green (505/515 nm), orange (540/560 nm), and deep red (633/660) dye coatings (FocalCheck fluorescence microscope test slide #1, slot A3, Thermo Fisher).
iii. Multicolor CSD-ISM characterization and Validation—for validating the resolution and color reconstruction we used subdiffraction limit, 170 nm fluorescent beads (green (505/515 nm), orange (540/560 nm), and deep red (633/660) PS-Speck microscope point source kit; Thermo Fisher). To prepare the slide, we mixed 2 μL bead solution of each color with the kit’s mounting medium and sonicated the mixture for 10 min. Then, 3 μL of the final mixture was placed on a microscope slide, air-dried for a few minutes, and covered with a clean 22 × 22 mm^2^ coverslip.
iv. Biological samples—for cell imaging we used primary mouse neuron cells expressing a mutant α-synuclein (α-Syn) associated with familial Parkinson’s disease via viral transduction. The neurons were prepared from D0-D1 mouse pups in accordance with the ethical guidelines of Tel Aviv University. Briefly, neurons cultured for 14 days and were fixated with 4% PFA, washed with 1× PBS, permeabilized with 0.1% Triton-X then blocked with bovine serum albumin (BSA) and normal goat serum (NGS). Next, neurons were incubated with primary antibodies G3BP (1:250 Abcam ab181150) and synaptotagmin-1­(1:500 synaptic systems #105001) at 4 °C overnight, followed by 1X PBS washes and then CF568 (1:1000 Bittium 20808) CF647 (1:1000 Bittium 20808) secondary antibody incubation for 2 h at room temperature. Lastly, washed cells were mounted on precleaned 1 mm thick microscopy slides with VectaShield DAPI mounting medium.

### Image Acquisition

Hardware configurations and image acquisition were managed using the Micro-Manager software.^46^ First, we confirmed the proper functioning of the CSD-ISM setup, resulting in a resolution improvement with a ratio of 1.45. This closely aligns with the theoretically expected value of approximately 1.41 (√2) [Section S2]. Next, the custom-designed linear Amici prism was integrated into our system, and consequently the DSP software parameters were adjusted for multicolor image acquisition. It was crucial to ensure that the spacing between adjacent confocal spots, controlled by the “Laser Pulse Number (LPN)” knob, allowed for dispersion without overlap. Additionally, the number of frames for image acquisition had to be set in both the DSP software GUI and Micro-Manager software. The effective frame rate of acquisition of 250 frames with CSD speed of 300 Hz, TC_ON_ = 100 and TC_OFF_ = 15 yields 0.01 Hz temporal resolution. This acquisition rate could be highly improved using better aligned lasers with higher excitation power densities.

Key acquisition parameters, including spinning disk rotation speed, laser pulse count, and the number of frames, were set based on the sample’s SNR and the number of color channels required for detection. In addition, by denoting the spinning disk period (single scan cycle) as TS, we established two constraints: TR = TS/LPN and TD = TR/N. The “Auto Rate” button in the DSP software GUI automatically adjusted these parameters to meet the acquisition requirements. The parameters for each experimetal phase were set as follows:

-Parameters for spectral calibration measurements: disk spinning speed was set to 1800 rpm (equivalent to the pulse rate of 360 Hz), and the following control parameters: TC_ON = 50–200 (optimized according to sample brightness and SNR), TC_OFF = 15, TL = 8 μs, TA = 0.0 μs, LPN = 2, *N* = 250.
-Parameters for image analysis testing, multicolor ISM validation and biological samples: the disk spinning speed was set to 1500 rpm (equivalent to the pulse rate of 300 Hz), and the following control parameters: TC_ON = 100, TC_OFF = 15, TL = 12 μs, TA = 0.0 μs, LPN = 2, N = 1000. For some images, we down-sampled the 1000 frames to 250 and 500 frames to simulate and benchmark shorter acquisition times.

All images featured in this study were subjected to adjustments in brightness and contrast using Fiji (ImageJ).^47^ Contrast enhancement was executed via Fiji’s “Enhance Contrast” function, employing a saturation threshold of 0.35%. Furthermore, the minimum display brightness was calibrated to 10% of the maximum pixel intensity to optimize visual clarity.

### Image Processing

All image analysis and the computation of the multicolor ISM image from the raw data stack were conducted using Python.^48^ For enhanced image resolution, the Richardson-Lucy (RL) deconvolution^37,38^ method was applied with 20 iterations, by employing the “Deconvolution Lab2”, RL Algorithm plugin within Fiji.^47^ The PSF for RL algorithm was determined experimentally by averaging the individual pinhole emission profiles acquired from a uniformly fluorescent dye (AF647) slide. This empirical PSF accounts for the specific optical characteristics and aberrations of our CSD-ISM architecture. Iteration counts were optimized using subdiffraction (170 nm) beads samples as a reference to ensure reconstruction fidelity and minimize the emergence of artifacts (for schematic diagram of image processing pipeline see Section S6).

i.  Spectral calibration and simulations—to calibrate the system’s spectral response, a series of steps were undertaken. Experimental PSF images from a range of fluorophores were collected (namely Cy5.5, AF647, AF568, AF546, AF532, AF488, and AF405), and theoretical emission spectra were sourced from Semrock’s SearchLight spectra viewer database .^49^ The calibration process involved fitting recorded intensity distributions to the theoretical center of mass wavelengths using a fourth-degree polynomial. This fit was employed to simulate PSFs for other fluorophores, accounting for optical components such as the dichroic mirror, emission filter, and camera’s spectral quantum efficiency. Using this calibration, we could simulate the spectral PSFs of additional fluorophores based on their theoretical emission spectra, which are readily available in online spectra-viewers^49–51^ (Section S2 and Figure S2).
ii. Localizing illumination spots’ positions—To register the illumination spots’ locations, for each experiment we acquired a single-color (AF647) reference stack with the same acquisition parameters as in the multicolor acquisition of the samples. The illumination spots’ locations were registered with subpixel precision using the “trackpy” Python package.^52^ These locations were used both for ISM pixel reassignment and spectral ROI cropping as shown in Figure 2.
iii. Spectral to color assignment—fluorophores present in the samples were selected, and their spectral PSFs were simulated. ROIs were then defined around each illumination spot, allowing for the cropping of spectral ROIs from the image stacks, thereby enhancing the accuracy of further analyses.
The spectral profiles for analysis and calibration were obtained from the Semrock’s SearchLight spectra viewer^49^ as follows:
- Image analysis validation—The spectra profiles of the 4 μm fluorescent beads are green-FocalCheck Double Green Ring (505/515), red—FocalCheck Red Ring (580/605), and far red—FocalCheck Dark Red Ring (633/660).
-Multicolor CSD-ISM characterization and validation—The spectra profiles of the 170 nm fluorescent beads are green-FluoSpheres Yellow-Green (505/515), orange—FluoSpheres Orange (580/605), and deep red—Alexa Fluor 633 (633/660).
-Biological sample—The following spectra profiles were used: nuclei—DAPI (359/461), green fluorescent protein GFP—eGFP (475/509), Ras GTPase-activating binding protein (G3BP)—CF568 (562/583) and synaptotagmin-1—CF647 (652/667).
iv. Spectral to color-channel decomposition—To decompose the spectral signatures into multicolor channels, ordinary least squares (OLS) regression was applied to the cropped ROIs. Each spectral signature within the cropped ROIs was decomposed into the simulated PSFs corresponding to the known sample labels through OLS regression. The output of the OLS analysis provided a set of three linear coefficients for each simulated PSF, indicating the composition of each PSF along with its two gradients based on their influence within the spectral ROI. To construct a color ROI, these triplet coefficients were linearly multiplied with the simulated PSF that most closely resembled a Gaussian profile (in this case, AF647). This process also incorporated gradient adjustments to account for minor parallax shifts, allowing for precise alignment. By utilizing the first Taylor approximation, the decomposition basis effectively enabled translation adjustments, thereby enhancing the accuracy of the reconstructed color image [see Section S3].
v. Color and ISM image reconstruction—Following the decomposition of spectral signatures, the next step involved color image reconstruction. After each ROI was reassigned both spatially and spectrally, the individual ROIs were summed and reconstructed to generate a multichannel composite image of the sample. To enhance resolution, the ISM reconstruction was applied based on the algorithm developed by Qin.^36^ Additional improvements were achieved through the RL-deconvolution algorithm, implemented via a Fiji plugin. This comprehensive approach facilitated the generation of final full-color and ISM images, allowing for improved clarity and detail in the visualization of the sample.

## Supplementary Material



## Data Availability

Data underlying the results presented in this paper are not publicly available at this time but may be obtained from the authors upon reasonable request. The entire analysis pipeline, together with example data sets, can be found in https://github.com/ebensteinLab.

## Notes

Optimization procedure and final parameters of prism design, spectral PSF simulation pipeline and results, and spectral-to-color channel decomposition algorithm and benchmarks results, flowchart of the analysis pipeline, resolution enhancement quantification and additional experimental data (PDF)

## ∇

Laboratory of Nanoscale Biology, Institute of Bioengineering (IBI); École Polytechnique Fédérale de Lausanne (EPFL), Lausanne 1015, Switzerland

## Abbreviations

ISM: image scanning microscopy
CSD: confocal spinning disk
DSP: sigital signal processor
FPGA: field-programmable gate array
GPU: graphics processing unit
PSF: point spread function
SIM: structured illumination microscopy
fwhm: full width at half maximum
SNR: signal-to-noise ratio
NA: numerical aperture
DAPI: 4′,6-diamidino-2-phenylindole
GFP: green fluorescent protein
AF: Alexa Fluor (a brand of fluorescent dyes)
CoCoS: continuously controlled spectral resolution
MSM: multifocal scanning microscopy
ROI: region of interest
FOV: field of view
OLS: ordinary least squares
